# Invasive Lionfish (*Pterois volitans*): A Potential Human Health Threat for Ciguatera Fish Poisoning in Tropical Waters

**DOI:** 10.3390/md12010088

**Published:** 2013-12-27

**Authors:** Alison Robertson, Ana C. Garcia, Harold A. Flores Quintana, Tyler B. Smith, Bernard F. Castillo, Kynoch Reale-Munroe, Joseph A. Gulli, David A. Olsen, Jennifer I. Hooe-Rollman, Edward L. E. Jester, Brian J. Klimek, Steven M. Plakas

**Affiliations:** 1 U.S. Food and Drug Administration, Division of Seafood Science and Technology, Gulf Coast Seafood Laboratory, 1 Iberville Drive, Dauphin Island, AL 36528, USA; E-Mails: agarci3@gmail.com (A.C.G.); Harold.FloresQuintana@fda.hhs.gov (H.A.F.Q.); Jennifer.Hooe-Rollman@fda.hhs.gov (J.I.H.-R.); Edward.Jester@fda.hhs.gov (E.L.E.J.); bklimek15@gmail.com (B.J.K.); Steven.Plakas@fda.hhs.gov (S.M.P.); 2 Department of Marine Sciences, University of South Alabama, 5871 University Drive North, Mobile, AL 36688, USA; 3 Center for Marine and Environmental Studies, University of the Virgin Islands, 2 John Brewers Bay, St. Thomas, VI 00802, USA; E-Mail: tsmith@live.uvi.edu; 4 College of Science and Mathematics, University of the Virgin Islands, RR1 Box 10000, Kingshill, VI 00850, USA; E-Mails: bcastil@live.uvi.edu (B.F.C.); kynoch_reale@yahoo.com (K.R.-M.); 5 The Caribbean Oceanic Restoration and Education (CORE) Foundation, Christiansted, VI 00824, USA; E-Mail: nolionfish@yahoo.com; 6 St. Thomas Fishermen’s Association, P.O. Box 308116, St. Thomas, VI 00803, USA; E-Mail: Olsen41@aol.com

**Keywords:** ciguatera fish poisoning, Caribbean ciguatoxins, lionfish, Caribbean, mass spectrometry

## Abstract

Invasive Indo-Pacific lionfish (*Pterois volitans*) have rapidly expanded in the Western Atlantic over the past decade and have had a significant negative impact on reef fish biodiversity, habitat, and community structure, with lionfish out-competing native predators for resources. In an effort to reduce this population explosion, lionfish have been promoted for human consumption in the greater Caribbean region. This study examined whether the geographical expansion of the lionfish into a known ciguatera-endemic region can pose a human health threat for ciguatera fish poisoning (CFP). More than 180 lionfish were collected from waters surrounding the US Virgin Islands throughout 2010 and 2011. Ciguatoxin testing included an *in vitro* neuroblastoma cytotoxicity assay for composite toxicity assessment of sodium-channel toxins combined with confirmatory liquid chromatography tandem mass spectrometry. A 12% prevalence rate of ciguatoxic lionfish exceeding the FDA guidance level of 0.1 µg/kg C-CTX-1 equivalents was identified in fish from the U.S. Virgin Islands, highlighting a potential consumption risk in this region. This study presents the first evidence that the invasive lionfish, pose a direct human health risk for CFP and highlights the need for awareness and research on this food safety hazard in known endemic areas.

## 1. Introduction

**Figure 1:**
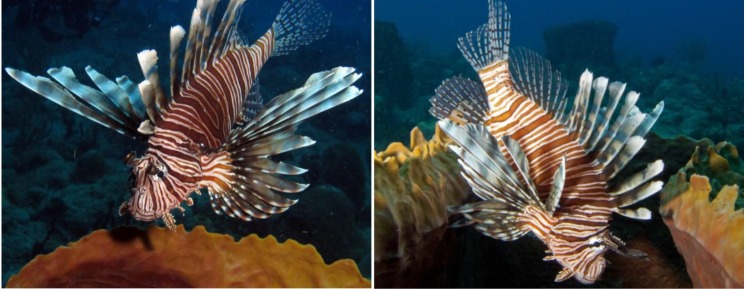
Lionfish (*P. volitans*) observed at “Grand Central Station” on the west coast of St. Croix.

Lionfish (*Pterois volitans*, Figure 1) are native to tropical and sub-tropical reef ecosystems in the southern Indian Ocean, South Pacific, and Red Sea [1]. With few natural predators, high reproductive rates, and high growth rates, lionfish have rapidly established populations in the northwestern Atlantic and Caribbean following their introduction into Florida waters in the 1990s [2,3]. In the U.S. Virgin Islands, lionfish were first reported off St. Croix in June 2008, and in St. Thomas and St. John in 2010 [2]. In less than four years, lionfish have become extremely abundant in the U.S. Virgin Islands. These population explosions have a dramatic ecological impact on reef fish biodiversity, habitat, and community structure, with lionfish out-competing native predators for resources [4,5,6]. In an effort to reduce their proliferation and geographical expansion into the Atlantic, Gulf of Mexico, and Caribbean Sea, lionfish have been identified as a fisheries resource, promoted in cookbooks and diving magazines, and served at fishing derbies and restaurants. While this could represent a great economic opportunity in local communities as an artisanal fishery, lionfish also pose a potential human health hazard as a vector for ciguatera fish poisoning (CFP) in endemic regions such as the US Virgin Islands. 

**Figure 2:**
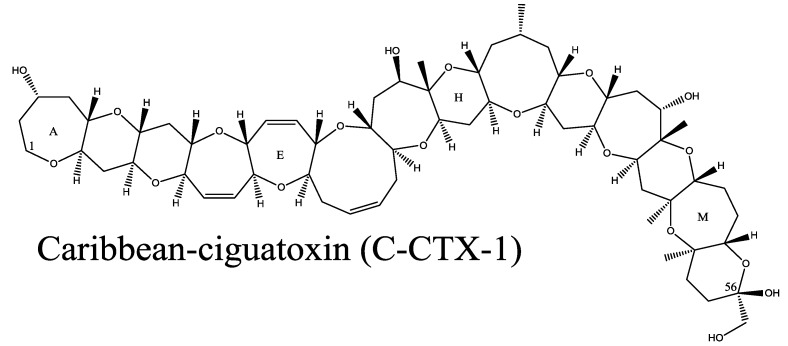
Structure of Caribbean ciguatoxin-1 (C-CTX-1). Caribbean ciguatoxin-2 (C-CTX-2) is an epimer of C-CTX-1 at carbon 56 noted on the structure.

CFP is a leading cause of seafood-borne illness and is estimated to cause up to 500,000 illnesses annually [7,8]. CFP is caused by the consumption of reef fish that have accumulated ciguatoxins (CTX, Figure 2). This acute poisoning syndrome is characterized by a variety of severe gastrointestinal, neurological, and occasionally cardiovascular symptoms that can occur within 4 h and last up to six weeks [9]. A chronic phase of CFP lasting many years has also been reported in up to 20% of those acutely exposed [10]. For acute CFP, the primary mode of action of CTX is via binding to site 5 on the voltage gated sodium channel in excitable tissues [11,12]. This slows down re-polarization and keeps channels in an open, depolarized state that can ultimately lead to cell death [12,13]. 

Precursors of CTX are produced by benthic dinoflagellates of the genus *Gambierdiscus.* These precursors, gambiertoxins, are taken up by herbivores grazing on the reef, and are converted to CTX during trophic transfer and metabolism in herbivorous and piscivorous fish [14]. Fish most commonly implicated in CFP include grouper, barracuda, snapper, jack, and mackerel [15]. CTX are tasteless, colorless, and odorless, therefore impossible to identify a toxic fish by sensory analysis [16]. Importantly, these toxins are stable under normal cooking temperatures and for extended periods of freezer storage [17]. To reduce the potential hazard of CFP, the U.S. Food and Drug Administration (FDA) recommends that primary processors obtain information about the harvest location and species of fish to determine the risk of contamination [15]. This study addresses the need for regional data on the potential consumption risk associated with CTX in lionfish.

## 2. Results and Discussion

**Figure 3:**
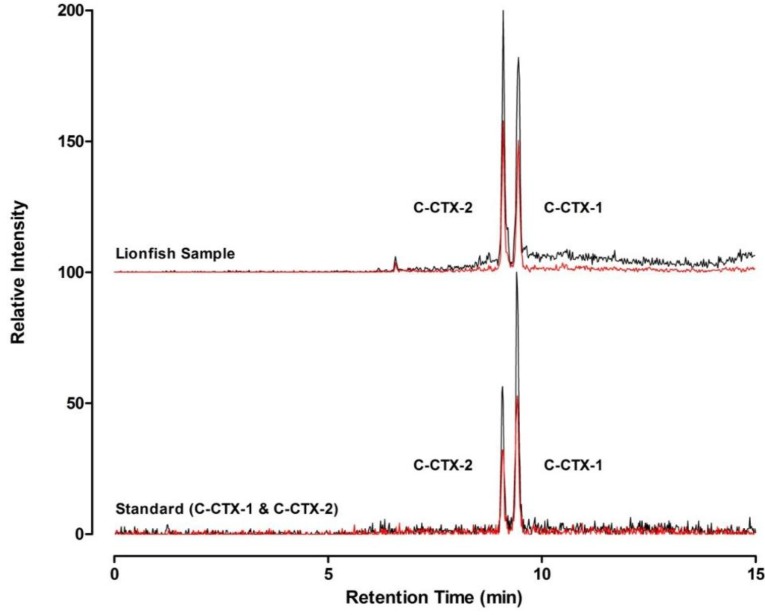
Confirmation of Caribbean ciguatoxins in lionfish flesh. Selected reaction monitoring (SRM) liquid chromatography mass spectrometry traces of C-CTX-1 and -2 in a lionfish flesh specimen (top) collected from St. Thomas compared to an authentic reference standard (bottom). The y-axes are offset for direct comparison. Two representative confirmatory transition ions (*m/z* 1123.7 > 1105.7, in black; and *m/z* 1123.7 > 1087.7, in red) are shown.

Of the 153 lionfish samples tested, 19 fish (St. Croix, *n* = 3; St. Thomas/St. John, *n* = 16) had a composite toxicity exceeding the FDA guidance level of 0.1 µg/kg C-CTX-1 equivalents and were confirmed to contain C-CTX-1 and -2 by LC-MS/MS (Figure 3). Additional C-CTX derivatives may contribute to the composite sodium channel specific toxicity in lionfish, but were not identified during this study. The highest toxicity level observed was 0.3 µg/kg C-CTX-1 equivalents in a lionfish collected from the south side of St. Thomas. Prevalence of lionfish samples above guidance levels was very similar between islands (St. Croix, 11.1%; St. Thomas/ St. John, 12.7%) Mean (±standard deviation) composite toxicity in lionfish tested above guidance was 0.19 ± 0.06 µg/kg C-CTX-1 equivalents. Fish size did not correlate to toxicity level. Forty-three lionfish (St. Croix, *n* = 5; St. Thomas/St. John, *n* = 38) tested positive by N2a assay at levels below the FDA guidance level. This represents an overall CTX prevalence rate of ~40% in lionfish collected from this region, and a ~12% prevalence rate for lionfish exceeding the FDA guidance level. The rates are comparable to other predatory reef fish, such as the schoolmaster snapper (*Lutjanus apodus*), which are avoided in fisheries where CFP is a known concern [18]. 

The concentrations of CTX detected in lionfish were most likely due to the location from which the fish were caught, length of time spent at that location, and the quantity of toxic prey consumed. The juvenile lionfish diet consists of mix of crustaceans and small teleost fish (3–10 g), with an increased reliance on fish as their size increases [3,19]. Common adult lionfish prey include representatives of the family Gobiidae, Labridae, Pomacentridae, Serranidae, and Blennidae [3,4], which are important vectors in the conversion of gambiertoxins originating from *Gambierdiscus*, into higher toxicity CTX congeners. It has been estimated that a single lionfish will consume over 50,000 fish per year [19], so the potential burden of CTX in this species would be expected to increase as a function of growth, size, and residence time on a toxic reef. Lionfish are highly localized, resident species within a given reef and thus bioaccumulation of CTXs through trophic transfer would be anticipated in areas harboring toxic *Gambierdiscus* spp. For these reasons, experience and local knowledge of safe fishing grounds remain the best strategy to avoid toxic fish. The limiting factor in this assessment would be the decline in available prey items for lionfish due to their negative impact on recruitment of other juvenile fish, and biodiversity [3,4]. 

## 3. Experimental Section

### 3.1. Reagents and Standards

HPLC grade acetone, methanol, hexane, chloroform, water, and acetonitrile were purchased from VWR (Suwanee, GA). All other reagents were purchased from Sigma Aldrich (St. Louis, MO, USA) and were of the highest grade available. Bond-elute silica solid phase extraction cartridges were sourced from Agilent (Santa Clara, CA, USA). Mouse neuroblastoma cells (Neuro-2a, CCL-131) were purchased from the American Type Culture Collection (Rockville, MD, USA). Whatman filter paper and all sterile cell culture consumables including serological pipettes, filter capped flasks, 96-well polystyrene plates, and culture flasks were purchased from Fisher (Suwanee, GA, USA). Culture media and heat inactivated fetal bovine serum were obtained from Life Technologies (Grand Is., NY, USA). All other cell supplements and reagents for assay including ouabain, veratridine, phosphate buffered saline, dimethyl sulfoxide, and 3-(4,5-dimethylthiazole-2-yl)-2,5-diphenyltetrazolium bromide (MTT) solution were obtained from Sigma Aldrich (St. Louis, MO, USA). C-CTX standards used in the cytotoxicity assay for composite toxicity of CTXs in lionfish were prepared at the FDA Gulf Coast Seafood Laboratory, Dauphin Island, AL. Additional C-CTX-1 standard was obtained from Richard Lewis, University of Queensland, Australia, and used along with FDA standards and reference materials in LC-MS confirmatory analyses. Purity was verified by LC-MS prior to use. 

### 3.2. Lionfish Sampling

**Figure 4:**
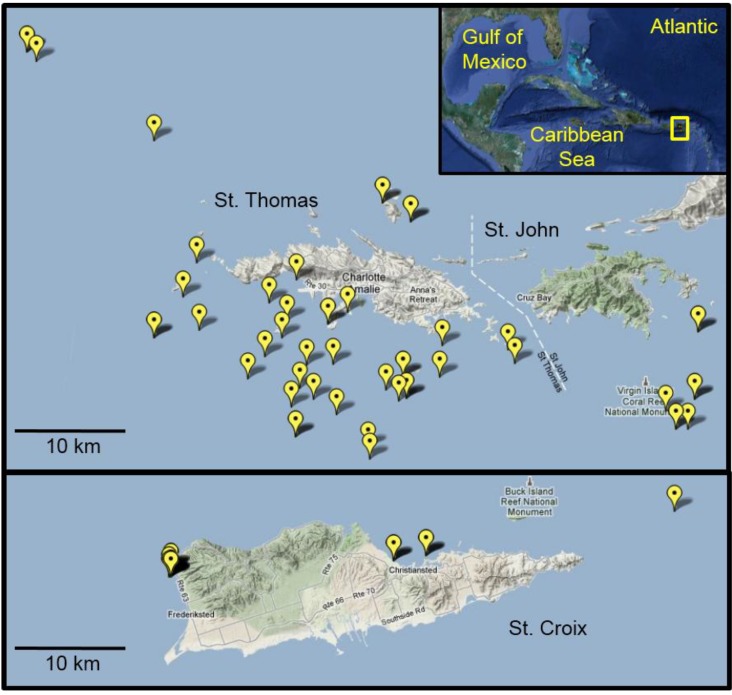
Lionfish collection sites in the U.S. Virgin Islands. Map details the global positioning system (GPS) coordinates from which lionfish were collected from August 2010 to December 2011. In many cases, multiple fish were collected from the individual sites. Inset shows an overview map of the Greater Caribbean region with the study area highlighted in yellow.

In Autumn 2010, we observed lionfish at our long-term monitoring sites in St. Thomas, an area hyperendemic for CFP [20]. These sites were established to examine the source and transmission of CTX in the marine food web in St. Thomas. With increasing reports of lionfish sightings and their harvest as a food source in this region, we expanded sampling efforts for this species to assess CFP risk. More than 180 lionfish were collected from the waters surrounding St. Thomas/St. John (*n* = 153) and St. Croix (*n* = 27) between September 2010 and December 2011 (Figure 4). Of these, all fish from St. Croix, and 126 lionfish from St. Thomas and St. John, were deemed large enough for analysis (>50 g body weight) and yielding sufficient flesh to be representative of an edible portion. Invasive lionfish were collected by divers on snorkel or SCUBA using a spear. Fish of a wide size range (50–600 g weight) were collected at depths between 2 and 60 m. The speared lionfish were euthanized underwater by cervical dislocation with a dive knife according to UVI and Institutional Animal Care and Use Committee (IACUC) recommendations for ecological depopulation field studies. Fish were stored in mesh collection bags during the remainder of the dive and returned to the surface and transferred to ice within 30 min of capture. Fish were frozen at −20 °C on return to the laboratory. Additional lionfish were collected by St. Thomas fishermen with West Indian fish traps (baited and unbaited) using standard commercial fishing practice for this region. Landed fish were subsequently frozen and shipped whole to the Gulf Coast Seafood Laboratory, Dauphin Island, AL, for processing, chemical extraction, and analysis. Scientific collection permits for lionfish were not required by the U.S. Virgin Islands Department of Planning and Natural Resources, Division of Fish and Wildlife, since these were an invasive species collected in non-restricted waters.

### 3.3. Sample Preparation and Toxin Extraction

Frozen whole lionfish were thawed to room temperature (~20 °C) and spines removed and discarded. Muscle tissue was subsequently filleted, and skin removed. All flesh harvested from an individual lionfish was passed through a meat grinder 2–3 times to homogenize the sample. From this homogenate, a sub-sample (30 to 100 g) was extracted with acetone (2 mL/g) in an explosion-proof stainless steel blender using standard FDA methodology [21]. Primary acetone extracts were filtered (Whatman #4) under vacuum, and tissue re-extracted in acetone in the same manner. Pooled, clarified, acetone extracts were then placed at −20 °C for at least 12 h to precipitate proteins. Extracts were filtered (Whatman #5) under vacuum, and filtrates dried by rotary evaporation. Dried residues were reconstituted in 80% aqueous methanol (1 mL/g tissue) and partitioned with *n*-hexanes (3 × 0.5 mL/g) to remove non-polar lipids. The aqueous methanolic phase was collected, dried, and residues reconstituted in water (1 mL/g tissue) and partitioned with chloroform (3 × 0.5 mL/g tissue). The pooled chloroform phases were dried and cleaned by silica solid phase extraction (SPE) according to previously reported FDA methods [21]. Standard spikes with a characterized C-CTX-1 reference were performed prior to extraction in blank lionfish tissue to assess extraction efficiency and recovery.

### 3.4. *In Vitro* Neuroblastoma Cytotoxicity Assay

CTX testing included an ouabain-veratridine dependent *in vitro* neuroblastoma cytotoxicity assay (N2a assay) for composite toxicity assessment of sodium-channel toxins [21,22]. Following chemical extraction of lionfish flesh and solid phase clean-up, all extracts were screened by N2a assay using standard protocols [21,22]. Neuro-2a cells were propagated and maintained in RPMI media supplemented with antibiotics (50 µg/mL streptomycin, 50 units/mL penicillin), glutamine (2 mM), sodium pyruvate (1 mM), and heat-inactivated FBS (10% v/v) as previously described [21]. Cells were harvested for assay when cultures were ~85%–90% confluent, and seeded at ~5 × 10^5^ cells/well into sterile 96-well polystyrene plates. When CTX activity was detected, full dose response curves (8-dilutions) of sample extracts were prepared to determine the concentration at which cell viability was reduced by 50% (ID_50_) compared with that of the CTX standard [21]. Aliquots (100 pg of toxin) of C-CTX-1 were used as a stock standard on each assay day. Results were expressed as µg/kg C-CTX-1 equivalents of fish tissue. All samples reported at 0.01 µg/kg total C-CTX-1 equivalents in fish tissue [15], were concentrated and analyzed by liquid chromatography tandem mass spectrometry (LC-MS/MS). 

### 3.5. Confirmatory Liquid Chromatography-Mass Spectrometry:

Sample extracts deemed positive for ciguatoxins by N2a assay were also analyzed by LC-MS/MS for unambiguous identification of CTX. This system consisted of a 1260 Agilent liquid chromatography system and Applied Biosystems MDS Sciex 4000 electrospray ionization quadrupole, linear ion trap, tandem mass spectrometer (Applied Biosystems, Inc., Foster City, CA, USA). Separation of the toxins was achieved on a Phenomenex Luna C18(2) analytical column (100 × 2 mm, 3 µM particle size) with a Phenomenex KrudKatcher ULTRA HPLC in-line filter (0.5 µm Depth × 0.004) both maintained at 40 °C. The mobile phase A consisted of 5 mM ammonium acetate in HPLC grade water (pH 5.4), and mobile phase B consisted of 5 mM ammonium acetate in 95% aqueous acetonitrile. Elution of confounding matrix components was achieved by initial gradient elution from 10% to 80% B over 6 min, then held at 80% B for isocratic separation of C-CTX congeners for an additional 6 min, before returning to the starting conditions. Flow rate was maintained at 0.3 mL/min. All analyses were performed in positive ion mode by selected reaction monitoring. Ion source parameters were as follows: source temperature, 400 °C; ion-spray voltage, 5000; nebulizer gas, 50 psi; curtain gas at 20 psi. Confirmation of C-CTXs was based on comparison with the retention time of reference standards (containing C-CTX-1 and C-CTX-2), and the presence of multiple ion transitions (corresponding to successive water losses) of the dehydrated parent [M + H − H_2_O]^+^ with a mass-to-charge ratio (*m/z*) of 1123.7 which fragmented to ions of *m/z* 1105.7, 1087.7, and 1069.7 at ratios consistent with standards. Compound specific parameters were optimized and were identical for C-CTX-1 and C-CTX-2 as follows: declustering potential at 100 V; entrance potential at 10 eV; Collision energy at 40; and, cell exit potential held at 15. A dwell time of 100 ms was applied for each transition ion pair with a resolution of unit/high quadrupole 1 and 3, respectively. Elution order of C-CTX-1 and C-CTX-2 was determined by in-line temperature stability and was consistent with previous data describing C-CTX-1 as the lower energy arrangement [17]. These analyses were possible due to our own stocks of purified C-CTX standards, however, with no commercial source of certified reference materials and considerable effort required for their preparation, these were used sparingly for the sensitive N2A assays and LC-MS confirmation. In future studies we hope to have sufficient standards to determine the toxicity equivalence factors and relative abundance of contributing CTXs in fish samples for LC-MS/MS quantification.

## 4. Conclusions

This study represents the first comprehensive data set documenting that lionfish are a vector of CTX in endemic regions. While no CFP illnesses associated with lionfish have been reported to FDA, 12% of the lionfish tested exceeded the FDA guidance level of 0.1 µg/kg C-CTX-1 equivalents, which highlights a potential human health risk for fish harvested from the U.S. Virgin Islands. The distribution of ciguatoxic fish can be sporadic even within a localized reef, so advice should be sought from experienced fishermen and local public health agencies prior to harvest. We have no evidence to suggest that lionfish collected from non-endemic areas would present a CFP risk. A multi-agency effort is now underway to further characterize the distribution and prevalence of toxic fish, and to assess the risks associated with the consumption of lionfish in other geographically relevant areas. 
